# Modifiable prognostic factors of high societal costs among people on sick leave due to musculoskeletal disorders: a replication study

**DOI:** 10.1186/s12891-024-08132-3

**Published:** 2024-12-03

**Authors:** Rikke Munk Killingmo, Tarjei Rysstad, Esther Maas, Are Hugo Pripp, Fiona Aanesen, Alexander Tingulstad, Anne Therese Tveter, Britt Elin Øiestad, Margreth Grotle

**Affiliations:** 1 Department of Rehabilitation Science and Health Technology, Oslo Metropolitan University, Oslo, Norway; 2 Department of Health Sciences, Faculty of Science, Vrije University Amsterdam, Amsterdam, The Netherlands; 3 The Amsterdam Movement Sciences Research Institute, Amsterdam, The Netherlands; 4 Oslo Centre of Biostatistics and Epidemiology Research Support Services, Oslo University Hospital, Oslo, Norway; 5 National Institute of Occupational Health, Oslo, Norway; 6 The Norwegian Medicines Agency, Oslo, Norway; 7 Center for Treatment of Rheumatic and Musculoskeletal Diseases (REMEDY), Diakonhjemmet Hospital, Oslo, Norway; 8 Department of Research and Innovation, Division of Clinical Neuroscience, Oslo University Hospital, Oslo, Norway

**Keywords:** Musculoskeletal disorders, Healthcare utilization, Productivity loss, Costs, Prognostic factor research

## Abstract

**Background:**

Musculoskeletal disorders are an extensive burden to society, yet few studies have explored and replicated modifiable prognostic factors associated with high societal costs. This study aimed to replicate previously identified associations between nine modifiable prognostic factors and high societal costs among people on sick leave due to musculoskeletal disorders.

**Methods:**

Pooled data from a three-arm randomised controlled trial with 6 months of follow-up were used, including 509 participants on sick leave due to musculoskeletal disorders in Norway. Consistent with the identification study, the primary outcome was societal costs dichotomised as high (top 25th percentile) or low. Societal costs included healthcare utilization (primary, secondary, and tertiary care) and productivity loss (absenteeism, work assessment allowance and disability benefits) collected from public records. Binary unadjusted and adjusted logistic regression analyses were used to replicate previously identified associations between each modifiable prognostic factor and having high costs.

**Results:**

Adjusted for selected covariates, a lower degree of return-to-work expectancy was associated with high societal costs in both the identification and replication sample.

Depressive symptoms and health literacy showed no prognostic value in both the identification and replication sample. There were inconsistent results with regards to statistical significance across the identification and replication sample for pain severity, self-perceived health, sleep quality, work satisfaction, disability, and long-lasting disorder expectation. Similar results were found when high costs were related to separately healthcare utilization and productivity loss.

**Conclusion:**

This study successfully replicated the association between return-to-work expectancy and high societal costs among people on sick leave due to musculoskeletal disorders. Other factors showed no prognostic value or inconsistent results.

**Trial registration:**

ClinicalTrials.gov NCT03871712, 12th of March 2019.

**Supplementary Information:**

The online version contains supplementary material available at 10.1186/s12891-024-08132-3.

## Background

Musculoskeletal disorders are highly prevalent and the leading cause of disability worldwide [1, 2]. The economic burden of musculoskeletal disorders is substantial, comprising both direct costs related to healthcare utilization and indirect costs related to productivity loss [3, 4]. In Norway, musculoskeletal disorders are a prevalent cause of healthcare utilization [5], and productivity loss [6, 7]. It is widely recognised that most of the socio-economic burden associated with musculoskeletal disorders stem from a relatively small subgroup of individuals [6–9]. Furthermore, there is a need to steer towards early identification and more cost-effective approaches for this subgroup [9–11]. Identifying and replicating modifiable prognostic factors associated with high societal costs is a crucial step for early identification and quality improvement of clinical practice [12]. Reliable information about these factors can guide development of targeted interventions, potentially enhancing both clinical effectiveness and cost efficiency [12]. Cost-effective interventions are essential to improve use of limited healthcare resources and alleviate the socio-economic burden of musculoskeletal disorders [10, 11, 13].

Individuals with high societal costs constitute a diverse population that appears to vary based on different health conditions, age groups, provider types (e.g., general practitioners, chiropractors, or hospitals), countries, and payer systems (e.g., government/public or private payer) [9, 14]. Therefore, generalisation of results cannot be done automatically. To the best of our knowledge, only one study [15] has investigated modifiable prognostic factors associated with high societal costs among a sample of people on sick leave due to musculoskeletal disorders. This study [15] identified pain severity, disability, self-perceived health, sleep quality, return-to-work expectancy, and long-lasting disorder expectation as modifiable prognostic factors of high societal costs. Replication of these findings is warranted [12, 15]. Therefore, the primary aim of this study was to replicate previously identified associations [15] between nine modifiable prognostic factors and high societal costs among people on sick leave due to musculoskeletal disorders. The secondary aim was to replicate previously identified associations [15] between the same factors and high costs related to separately 1) healthcare utilization, and 2) productivity loss.

## Method

This study was planned and conducted in accordance with the PROGnosis RESearch Strategy (PROGRESS) framework [12, 16]. A study protocol [17] has been published, and a statistical analysis plan for this secondary analysis [18] was registered in the original trial registration prior to analysis. The REporting recommendations for tumour MARKer prognostic studies (REMARK) criteria [19] were followed.

### Design and setting

This study includes a secondary analysis embedded in a three-arm, pragmatic randomised controlled trial (RCT) with 6 months of follow-up, conducted within the Norwegian Labour and Welfare Administration (NAV); the work package three of the MI-NAV project [17].

### Participants, recruitment procedure, stratification, and randomisation

The identification study consisted of 549 workers (aged 18–67 years) on sick leave (full or partial, ≥ 4 consecutive weeks) due to musculoskeletal disorders in Norway (the work package two of the MI-NAV project [15]).

Eligible participants for the replication study were workers, aged 18 to 67 years, on sick leave (50–100% sick leave rate, ≥ 7 consecutive weeks) due to musculoskeletal disorders. Exclusion criteria were serious somatic or mental health disorders affecting work ability and entailing specialised treatment (e.g., cancer, psychotic disorders), pregnancy, unemployed, freelancers and self-employed workers, and insufficient Norwegian or English language skills to answer questionnaires or communicate by telephone. Participants were recruited through a phone call from NAV between April 2019 and October 2020. All participants provided electronic informed consent prior to study enrolment and were informed of their right to withdraw at any time.

The Örebro Musculoskeletal Pain Screening Questionnaire Short Form (ÖMPSQ-SF) [20] and the Keele STarT MSK Tool (STarT MSK) [21] were used to stratify participants into risk groups of long-term sick leave [17, 22]. After stratification, participants were randomly allocated to either usual case management for people on sick leave in Norway (UC), motivational interviewing (MI) and UC, or a stratified vocational advice intervention (SVAI) and UC, with a 1:1:1 allocation within each stratum of low/medium and high-risk. In this study, all included participants were pooled into one sample.

### Interventions

A comprehensive description of the rationale, development and content of the interventions can be found elsewhere [17, 22]. Briefly, all participants were offered UC. In addition, participants in the MI arm were offered two face-to-face sessions of MI from a NAV caseworker. Participants in the SVAI arm were offered vocational advice and case management from physiotherapists. Those stratified to the low/medium-risk group were offered 1–2 telephone sessions. Participants in the high-risk group were offered 3–4 sessions.

### Data collection, outcome, modifiable prognostic factors, and covariates

At baseline, all participants completed an electronic questionnaire including demographic variables and a number of patient-reported measures. Data on healthcare utilization was obtained from public records including the Norwegian Patient Registry (NPR) and the Municipal Patient and User Registry (KPR). Data on productivity loss was also obtained from public records (NAV), which detailed dates and grading of absenteeism, work assessment allowance, and disability benefits, as well as the related diagnostic code, and contracted workhours. These data on healthcare utilization and productivity loss were obtained in the period from baseline to 3 months retrospectively, and during the 6 months follow-up period. To assess representativeness of the study sample, we collected anonymised registry data covering sex, age, occupation, and contracted work hours from all eligible candidates. All data were stored and analyzed securely using the Service for sensitive data (TSD) [23].

#### Outcomes

Aside from the duration of follow-up (12 months in the identification study, 6 months in this replication study), outcomes were identical to those outlined in the identification study [15]. The primary outcome was societal costs aggregated for 6 months of follow-up and categorised as either high or low. High costs were defined as being in the top 25th percentile of costs [24, 25]. Healthcare utilization included primary healthcare use (general practitioner (GP), physiotherapist, chiropractor, and emergency room consultations), as well as secondary/tertiary healthcare use (outpatient contacts, day surgery, ordinary admission with overnight stay, and other admissions without overnight stay). Productivity loss included absenteeism, work assessment allowance, and disability benefits. Costs of healthcare utilization per person were estimated by reimbursement rates obtained from NPR and KPR. Costs of productivity loss per person were estimated by multiplying number of days with productivity loss, adjusted for employment rate and grading of productivity loss, by an estimated average wage rate (from official statistics in Norway) including taxes and social costs.

Secondary outcomes were costs related to separately 1) healthcare utilization aggregated for 6 months of follow up and categorised as high or low, and 2) productivity loss aggregated for 6 months of follow up and categorised as high or low.

#### Modifiable prognostic factors

Modifiable prognostic factors were identical to those outlined in the identification study [15], and included the following self-reported variables measured at baseline: pain severity measured by the Numeric Rating Scale (NRS) (range 0–10, higher score indicating higher pain severity) [26]; disability measured by a single item (Q3) from the EuroQol 5 dimensions (EQ-5D-5L) [27] and re-categorised into no/slight problems, moderate problems or severe problems/unable to do; self-perceived health measured by a single item (EQ VAS) from the EQ-5D-5L (range 0–10, reversed score, higher score indicating poorer health) [27]; depressive symptoms measured by a single item (Q6) from the ÖMPSQ-SF (range 0–10, higher score indicating more depression symptoms) [20]; sleep quality measured by a single item (Q4) from the ÖMPSQ-SF (range 0–10, reversed score, higher score indicating poorer sleep quality) [20]; health literacy measured by a single item (Q12) from the Musculoskeletal Health Questionnaire (MSK-HQ) [28] and re-categorised into completely/very well understanding, moderate understanding or slightly/no understanding; work satisfaction measured by a single item (0–10, higher score indicating lower work satisfaction); long-lasting disorder expectation measured by a single item (Q6) from the STarT MSK tool [21] and categorised into yes/no; and return-to-work expectancy measured by a single item (Q8) from ÖMPSQ-SF (range 0–10, reversed score, higher score indicating lower return-to-work expectancy) [20].

#### Covariates

As in the identification study [15], we adjusted for potential covariates when evaluating the modifiable prognostic factors. Potential covariates were identical to those in the identification study [15], and included the following self-reported variables measured at baseline: sex (female/male); age (years); education level categorised as low or high (university level); and pain duration assessed by a single item (Q1) from the ÖMPSQ-SF [20] and categorised as < 3 months or ≥ 3 months. Also, the following variables from public records were included as covariates: absenteeism-related diagnosis type at baseline collected from NAV and categorised into “upper/lower limb conditions”, “back/neck conditions”, “fractures”, “multisite pain/joint conditions”, or “other MSK conditions”; total healthcare utilization costs for the 3 months prior to inclusion, estimated from NPR and KPR; and total productivity loss costs for the 3 months prior to inclusion, estimated from NAV. In addition, group allocation was included as a potential covariate [29].

### Analyses

All analyses were performed in accordance with the statistical analysis plan published a priori [18] using the IBM SPSS version 29 (IBM Corporation, Armonk, NY, USA) and Stata version 17.0 (StataCorp LLC, College Station, TX). No correction for multiple testing was performed and two-sided p-values < 0.05 were deemed statistically significant.

We visually explored missing value patterns, and missingness was assumed to be at random. Additionally, evidence was found against the hypothesis that values were not missing completely at random (Little’s test, p > 0.05). Missing data for the included baseline variables ranged from 0.0 to 1.8%, with no missing data for variables used in calculating outcome scores. Given the low proportion of missing data (0.1%), and minimal differences between responders and non-responders, complete case analyses were conducted.

Calculation of healthcare utilization, productivity loss and cost estimation were consistent with methodology used in the identification study [15]. Type and frequency of healthcare utilization were calculated per person for the 6 months of follow-up period. Days of productivity loss were calculated per person for the same period, adjusted for employment rate and grading of productivity loss. Costs of healthcare utilization per person were estimated using reimbursement rates from NPR and KPR. Non-healthcare costs related to healthcare provision, such as transportation, were not included. Costs of productivity loss per person were estimated by multiplying number of days with complete productivity loss by an estimated average wage rate from official Norwegian statistics, including taxes and social costs: absenteeism was set at €343 per workday, work assessment allowance and disability benefits were set at €227 per workday. All costs were converted to euros (€) 2022 and presented as both mean and median values, including 95% confidence intervals (CIs), using bias-corrected and accelerated (BCa) bootstrapping with 1000 simulations. Norwegian prices were converted to euros based on the January 2022 exchange rate (€1 = NOK 10).

Binary logistic regression models were used to replicate findings from the identification study [15]. Associations (crude and adjusted for selected covariates) between each modifiable prognostic factor and total costs related to 1) healthcare utilization and productivity loss, 2) healthcare utilization, and 3) productivity loss were assessed. As in the identification study [15], the cost score was entered into the model as a dependent dichotomous variable, with high cost defined as participants with cost in the top 25th percentile (yes/no). Linearity of continuous independent variables were assessed using the multivariable fraction polynomial method [30]. Aside from “sleep quality” and “total costs related to healthcare utilization prior to inclusion”, all continuous independent variables demonstrated a linear relationship with the outcomes. The “sleep quality” variable was modelled using multivariable fraction polynomial 1 before incorporated into the analyses. The “total cost related to healthcare utilization prior to inclusion” variable was incorporated as a linear component in the analyses due to sample size constrains and its role solely as a covariate in one of the secondary analyses. The results were presented as crude and adjusted odds ratios (OR) with 95% CI. The decision on whether findings from the identification study are replicated were based on the direction and size of the association, as well as the confidence interval for each of the predefined modifiable prognostic factors [31].

#### Sensitivity analysis

To evaluate credibility of the societal cost calculation in the main analyses, the calculation was performed without outliers. Consistent with the methodology used in the identification study [15], outliers were identified through simple scatterplots by visual inspection and defined as participants with remarkably high societal costs. To assess the effect of not modelling the “total cost related to healthcare utilization prior to inclusion” variable in the main analyses, the variable was modelled using multivariable fraction polynomial 1 before incorporated into the analyses.

#### Sample size

This study had a fixed sample size. To determine statistical power, we applied the number of events per parameter (EPP) method [32–36] and the rule-of-thumb of “10 events per parameter included” [37–40]. With a sample size of 509 participants, we expected 127 participants to be within the top 25th percentile of costs and categorised as having high costs (yes/no) (events). An EPP of 10 permits for a maximum of 13 parameters to be included in the final multivariable prediction model.

## Results

A total of 509 participants were eligible for analysis. Flow of participants through the RCT is shown elsewhere [22]. Table 1 presents participants characteristics and clinical status at baseline, as well as the proportion of missing data per variable, in both the identification and replication sample. Largely, the samples were balanced. Yet, the replication sample had a somewhat lower proportion of participants with a full-time employment status (66% vs 77%), and who contacted a physiotherapist prior to inclusion (27% vs 39%) compared to the identification sample. Furthermore, a higher proportion of participants in the replication sample had a pain duration period of less than 3 months (40% vs 21%), and an emergency consultation prior to inclusion (20% vs 8%) compared to the identification sample.

**Table 1:** Baseline characteristics of the study participants in the identification sample and the replication sample

	Identification sample		Replication sample	
	All participants (*n* = 549)	Missing, n (%)	All participants (*n* = 509)	Missing, n (%)
Female, n (%)	309 (56)	0 (0)	291 (57)	0 (0)
Age in years, median (IQR)	50 (42–57)	0 (0)	49 (41–55)	0 (0)
Education at university level, n (%)	220 (40)	1 (0.2)	187 (37)	0 (0)
Mother tongue Norwegian, n (%)	473 (87)	2 (0.4)	448 (88)	2 (0.4)
Employment status, n (%)		0 (0)		0 (0)
Full-time	420 (77)		338 (66)	
Part-time, 50–99% of full-time position	101 (18)		139 (27)	
Part-time, < 50% of full-time position	28 (5)		32 (7)	
Diagnosis (ICPC-2)*, n (%)		0 (0)		9 (2)
Upper limb conditions	121 (22)		90 (18)	
Lower limb conditions	47 (9)		39 (8)	
Neck conditions	36 (7)		34 (7)	
Back conditions	107 (19)		119 (24)	
Multisite pain/joint conditions	54 (10)		73 (14)	
Fractures/injury/trauma	51 (9)		41 (8)	
Other MSK conditions	133 (24)		104 (21)	
Pain severity average last week (NRS, 0–10), mean (SD)	6 (2)	0 (0)	6 (2)	0 (0)
Pain duration, n (%)		0 (0)		0 (0)
< 3 months	114 (21)		205 (40)	
3–6 months	87 (16)		68 (13)	
> 6 months	348 (63)		236 (47)	
Disability (EQ-5D-5L, Q3), n (%)		1 (0.2)		2 (0.4)
No problems doing usual activities	36 (6)		23 (5)	
Slight problems doing usual activities	173 (32)		164 (32)	
Moderate problems doing usual activities	200 (36)		185 (36)	
Severe problems doing usual activities	119 (22)		116 (23)	
Unable to do usual activities	20 (4)		19 (4)	
Self-perceived health (EQ-5D-5L, EQ VAS, 0–10)^a^, mean (SD)	5 (2)	7 (1)	5 (2)	2 (0.4)
Depressive symptoms (ÖMPSQ-SF, Q6, 0–10)^a^, mean (SD)	3 (3)	0 (0)	3 (3)	0 (0)
Sleep quality (ÖMPSQ-SF, Q4, 0–10)^a^, mean (SD)	5 (3)	0 (0)	5 (3)	0 (0)
Long-lasting disorder expectation (STarT MSK, Q6), n (%)	448 (82)	0 (0)	411 (81)	0 (0)
Health literacy (MSK-HQ, Q12), n (%)		0 (0)		1 (0.2)
Completely understanding of condition/treatment	75 (14)		75 (15)	
Very well understanding of condition/treatment	268 (49)		234 (46)	
Moderately understanding of condition/treatment	134 (24)		134 (26)	
Slightly understanding of condition/treatment	55 (10)		51 (10)	
No understanding of condition/treatment	17 (3)		14 (3)	
Return-to-work expectancy (ÖMPSQ-SF, Q8, 0–10)^a^, mean (SD)	4 (3)	0 (0)	3 (3)	0 (0)
Work satisfaction (0–10)^a^, mean (SD)	2 (3)	9 (2)	3 (2)	1 (0.2)
Healthcare utilization prior to inclusion**				
Primary care consultation last 3 months, n (%)		0 (0)		0 (0)
General practitioner	539 (98)		503 (99)	
Physiotherapist	216 (39)		137 (27)	
Chiropractor	68 (12)		71 (14)	
Emergency room	45 (8)		101 (20)	
Secondary/tertiary care last 3 months, n (%)		0 (0)		0 (0)
Outpatient contact	203 (37)		208 (41)	
Day surgery	30 (6)		35 (7)	
Ordinary admission with overnight stay	28 (5)		61 (12)	
Other admissions without overnight stay	46 (8)		31 (6)	
Productivity loss prior to inclusion***				
Days of sick leave last 3 months, median (IQR)	30 (21–43)	0 (0)	31 (22–36)	0 (0)
Days of work assessment allowance last 3 months, median (IQR)	0 (0)	0 (0)	0 (0)	0 (0)
Days of disability benefits last 3 months, median (IQR)	0 (0)	0 (0)	0 (0)	0 (0)

EQ-5D-5L indicates EuroQol 5 dimensions, *ICPC-2* International Classification of Primary Care 2ed edition, *IQR* interquartile range, *MSK-HQ* Musculoskeletal Health Questionnaire, *n* number of individuals/observations, *NRS* Numeric Rating Scale, *ÖMPSQ-SF* Örebro Musculoskeletal Pain Screening Questionnaire Short Form, *SD* standard deviation, *STarT MSK* Keele STarT MSK tool; *%* percentage

^a^A lower score is better

^*^Absenteeism related diagnoses type collected from the Norwegian Labour and Welfare Administration (NAV) registry

^**^Collected from public records; the Norwegian Patient Registry (NPR) and the Municipal Patient and User Registry (KPR)

^***^Collected from the NAV registry, measured as calendar days, and adjusted for employment rate and grading of productivity loss. All percentage numbers are presented as valid percentage of total

### Healthcare utilization, productivity loss and cost estimation

Table 2 provides aggregated data on healthcare utilization and productivity loss for the 6 months of follow-up period. Table 3 details total costs related to healthcare utilization and productivity loss for the same period. Consistent with the identification study [15], costs were predominantly driven by productivity loss, which accounted for 91% of total costs during the 6 months of follow-up. A total of 127 participants (25%) were classified as having high costs related to healthcare utilization and productivity loss (≥ €34 078), healthcare utilization (≥ €2125), and productivity loss (≥ €31 445). Two participants (0.4%) were defined as outliers due to healthcare utilization costs exceeding €16 575. The two outliers had costs related to primary, secondary, and tertiary healthcare use.

**Table 2:** Healthcare utilization and productivity loss aggregated for six months of follow-up

	All participants (*n* = 509)	Missing, n (%)
*Primary care*		
Participants with primary care consultation, n (%)		0 (0)
General practitioner	499 (98)	
Physiotherapist	202 (40)	
Chiropractor	72 (14)	
Emergency room	129 (25)	
No primary care consultation	4 (1)	
Numbers of consultations, median (IQR)^a^		0 (0)
General practitioner	9 (6–13)	
Physiotherapist	7 (3–17)	
Chiropractor	4 (2–9)	
Emergency room	2 (1–2)	
*Secondary/tertiary care*		
Participants with secondary/tertiary care consultation, n (%)		0 (0)
Outpatient contact	324 (64)	
Day surgery	24 (5)	
Ordinary admission with overnight stay	39 (8)	
Other admissions without overnight stay	78 (15)	
No secondary/tertiary care consultation	165 (32)	
Numbers of consultations, median (IQR)*		0 (0)
Outpatient contact	2 (1–3)	
Day surgery	1 (1–1)	
Ordinary admission with overnight stay	1 (1–1)	
Other admissions without overnight stay	1 (1–3)	
Duration of ordinary admission with overnight stay in days, median (IQR)^b^	1 (1–2)	
*Productivity loss*		
Participants with productivity loss, n (%)		0 (0)
Sick leave	505 (99)	
Work assessment allowance	12 (2)	
Disability benefits	0 (0)	
Duration of productivity loss in days, median (IQR)^c^		0 (0)
Sick leave	55 (25–89)	
Work assessment allowance	0 (0–0)	
Disability benefits	0 (0–0)	

IQR indicates interquartile range; n, number of individuals/observations; %, percentage

^a^Calculated on basis of participants who have reported primary/secondary/tertiary care consultations

^b^Calculated on basis of participants who have reported ordinary admission with overnight stay

^c^Calculated on basis of participants who have reported productivity loss, converted into a 5-day workweek, and adjusted for employment rate and grading of productivity loss

**Table 3:** Cost (€) due to healthcare utilization and productivity loss aggregated for six months of follow-up (*n* = 509)

	Participants with zero cost, n (%)	Mean costs (95%CI^a^)	Median costs (95% CI^a^)
Primary care			
General practitioner	10 (2)	552 (515—588)	473 (431—514)
Physiotherapist	307 (60)	321 (260—381)	0 (0—0)
Chiropractor	437 (86)	56 (40—72)	0 (0—0)
Emergency room	380 (75)	25 (20—31)	0 (0—0)
Total primary care	4 (1)	954 (875—1 033)	678 (620—735)
Secondary/tertiary care			
Outpatient contact	185 (36)	293 (258—328)	205 (180—231)
Day surgery	485 (95)	96 (57—134)	0 (0—0)
Ordinary admission with overnight stay	470 (92)	572 (362—782)	0 (0—0)
Other admissions without overnight stay	431 (85)	102 (67—137)	0 (0—0)
Total secondary/tertiary care	165 (32)	1 062 (834—1 290)	239 (201—276)
Productivity loss			
Sick leave	4 (1)	20 321 (19 147—21 495)	18 637 (16 011—21 263)
Work assessment allowance	495 (97)	264 (79—449)	0 (0—0)
Disability benefits	509 (100)	0 (0—0)	0 (0—0)
Total productivity loss	3 (1)	20 584 (19 420—21 748)	19 445 (16 906—21 984)
**Total**	1 (0.2)	22 600 (21 356—23 845)	21 145 (18 927—23 363)

Cost related to productivity loss are calculated on basis of reported days with productivity loss, converted into a 5-day workweek, and adjusted for employment rate and grading of productivity loss

^a^Bias-corrected and accelerated bootstrapping (1000 simulations)

### Replication analysis

Table 4 presents crude and adjusted OR with 95% CI for the association between each of the modifiable prognostic factor and being in the three high costs groups. Out of nine modifiable prognostic factors, only a lower degree of “return-to-work expectancy” showed a statistically significant crude and adjusted association with an increased odds of being in the high societal costs group. Similar results were found for the secondary outcomes. Additionally, a lower degree of “work satisfaction” demonstrated a statistically significant association with decreased odds of being in the high healthcare utilization costs group, while a long-lasting disorder expectation was statistically significant association with increased odds of being in the high productivity loss costs group. The sensitivity analysis (Tables 1 and 2 in the Additional file 1) provided similar results, when comparing complete case analysis and analysis where the “total costs related to healthcare utilization prior to inclusion” variable was modelled with multivariable fraction polynomial 1 to the main analysis.

**Table 4:** Binary logistic regression analyses; individual associations between modifiable prognostic factors and high societal costs, high cost related to healthcare utilization, and high cost related to productivity loss (*n* = 509)

	**High societal costs**		**High costs related to healthcare utilization**		**High costs related to productivity loss**	
	Crude OR (95% CI)	Adjusted OR* (95% CI)	Crude OR (95% CI)	Adjusted OR* (95% CI)	Crude OR (95% CI)	Adjusted OR* (95% CI)
Pain severity (NRS, 0–10)	1.05 (0.95–1.16)	1.09 (0.97–1.23)	1.00 (0.91–1.11)	1.04 (0.93–1.16)	1.05 (0.95–1.17)	1.08 (0.96–1.22)
Self-perceived health (EQ-5D-5L, EQ VAS, 0–10)	1.07 (0.97–1.19)	1.06 (0.95–1.20)	1.06 (0.96–1.18)	1.07 (0.96–1.19)	1.11 (0.99–1.23)	1.10 (0.98–1.24)
Depressive symptoms (ÖMPSQ-SF, Q6, 0–10)	0.96 (0.90–1.03)	0.96 (0.89–1.03)	1.01 (0.94–1.08)	1.02 (0.95–1.09)	0.98 (0.92–1.05)	0.97 (0.90–1.05)
Sleep quality (ÖMPSQ-SF, Q4, 0–10)^a^	1.06 (0.98–1.14)	1.07 (0.98–1.16)	1.04 (0.97–1.13)	1.05 (0.97–1.14)	1.06 (0.98–1.14)	1.09 (0.99–1.18)
Return-to-work expectancy (ÖMPSQ-SF, Q8, 0–10)	1.20 (1.12–1.30)	1.22 (1.12–1.33)	1.08 (1.00–1.16)	1.09 (1.00–1.18)	1.20 (1.12–1.29)	1.23 (1.12–1.34)
Work satisfaction (0–10)	1.03 (0.95–1.11)	1.01 (0.93–1.11)	0.87 (0.80–0.96)	0.89 (0.80–0.98)	1.01 (0.93–1.09)	0.98 (0.89–1.08)
Disability (EQ-5D-5L, Q3) (ref: no/slight problems)						
Moderate problems	0.78 (0.48–1.25)	0.78 (0.46–1.33)	1.17 (0.73–1.88)	1.22 (0.74–2.00)	0.73 (0.45–1.18)	0.74 (0.43–1.29)
Severe problems/unable to do	1.10 (0.67–1.81)	1.01 (0.59–1.75)	1.22 (0.73–2.03)	1.12 (0.66–1.91)	1.19 (0.72–1.94)	1.11 (0.64–1.96)
Health literacy (MSK-HQ, Q12) (ref: completely/very well understanding)						
Moderate understanding	0.98 (0.61–1.57)	0.98 (0.58–1.66)	0.77 (0.48–1.25)	0.85 (0.52–1.41)	1.16 (0.73–1.86)	1.12 (0.66–1.91)
Slightly/no understanding	1.29 (0.71–2.34)	1.02 (0.52–1.99)	0.67 (0.35–1.29)	0.69 (0.34–1.40)	1.36 (0.75–2.47)	1.11 (0.56–2.20)
Long-lasting disorder expectation (STarT MSK, Q6) (ref: no)	1.60 (0.92–2.80)	1.49 (0.80–2.79)	1.48 (0.86–2.56)	1.47 (0.81–2.68)	1.90 (1.06–3.38)	1.94 (1.01–3.76)

EQ-5D-5L indicates EuroQol 5 dimensions; *CI* confidence interval; *MSK-HQ* Musculoskeletal Health Questionnaire; *NRS* Numeric Rating Scale; *OR* odd ratio; *ÖMPSQ-SF* Örebro Musculoskeletal Pain Screening Questionnaire Short Form; *STarT MSK* Keele STarT MSK tool; *Q* question number

^a^Fractional polynomial function = Sleep quality −4.5914

^*^Adjusted by sex, age, education level, absenteeism related diagnosis type, pain duration, group allocation, and costs related to 1) healthcare utilization and productivity loss prior to inclusion, 2) healthcare utilization prior to inclusion, or 3) productivity loss prior to inclusion

**Fig. 1:**
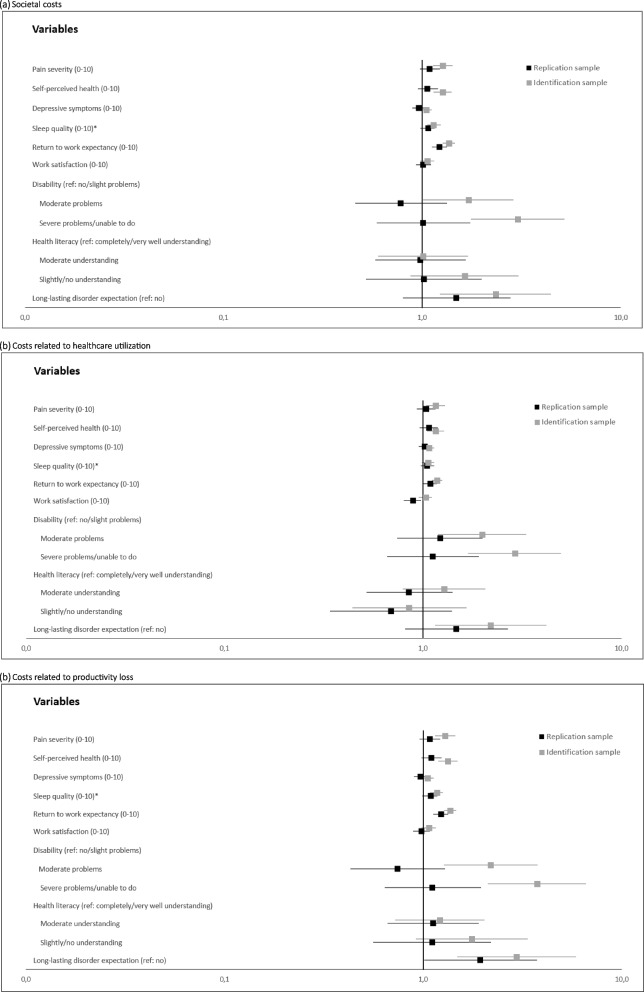
Forest plot summary of binary logistic regression analyses for individual associations between modifiable prognostic factors and high costs related to (**a**) healthcare utilization and productivity loss, (**b**) healthcare utilization, and (**c**) productivity loss. Adjusted (by sex, age, education level, absenteeism related diagnosis type, pain duration, group allocation (replication study), and costs related to (**a**) healthcare utilization and productivity loss prior to inclusion, (**b**) healthcare utilization prior to inclusion, or (**c**) productivity loss prior to inclusion) odds ratios (boxes) and the corresponding 95% confidence intervals (lines) are shown in black and grey for the replication and identification study, respectively. *Fractional polynomial function was used in the external validation study = Sleep quality −4.5914

Figure 1 shows a summary of adjusted OR estimates with 95% CI for the association between each of the modifiable prognostic factors and being in one of the three high costs groups, for both the identification and replication sample. Except for depressive symptoms, the disability “moderate problems” group, and the health literacy “moderate understanding” group, findings from the identification study [15] were replicated with respect to the direction of the adjusted association between each of the prognostic factors and the primary outcome. Though, the magnitude of the association varied > 20% for the following factors: disability “severe problems/unable to do” group, health literacy “slightly/no understanding” group, and long-lasting disorder expectation. In both the identification [15] and replication analysis, after adjustment for selected covariates and with the “low cost group” as the reference, only a lower degree of return-to-work expectancy was statistically significantly associated with increased odds of being in one of the three high costs groups. In both analyses, no association was found between being in one of the three high costs groups and the degree of depressive symptoms or health literacy.

## Discussion

This study aimed to replicate previously identified associations between nine modifiable prognostic factors and high societal costs among people on sick leave due to musculoskeletal disorders. Return-to-work expectancy was replicated as a statistically significant modifiable prognostic factor of high societal costs. Depressive symptoms and health literacy showed no prognostic value in both the identification and replication study. There were inconsistent results regarding statistical significance across the two studies with regards to pain severity, self-perceived health, sleep quality, work satisfaction, disability, and long-lasting disorder expectation. However, the direction of the adjusted association between each of these six factors and high societal costs was replicated, except for the disability “moderate problems” group, and the health literacy “moderate understanding” group. More or less similar results were found when high costs were related to separately healthcare utilization and productivity loss.

To the best of our knowledge, no similar studies have focused exclusively on a sample of people on sick leave due to musculoskeletal disorders, limiting direct comparability of our findings with other studies. Nonetheless, our findings are generally in accordance with existing scientific literature on patients with back pain [14, 24, 25, 41–48], as well as people with musculoskeletal disorders [49–54] and non-diagnosis-specific studies [55–57]. Our finding showing the impact of return-to-work expectancy on societal costs align with previous research [48, 57, 58]. Similarly, our findings regarding depression symptoms are consistent with previous research. Although several studies [14, 24, 25, 44, 45, 47, 51, 57] have identified depression symptoms as being associated with both healthcare utilization and productivity loss, a systematic review by Steenstra et al. [48] concluded that depression symptoms were not a prognostic factor of return-to-work in the later phases of work disability (> 6 weeks). Our findings, which did not replicate pain severity, self-perceived health, sleep quality, work satisfaction, disability, and long-term disorder expectations as prognostic factors of high societal costs in the later phases of work disability (> 7 weeks), also align with previous research. Although these factors are supported by strong evidence in the early phases [14, 24, 25, 41–47, 49–53, 55–57], the evidence is less clear in the lather phases of work disability [48]. This discrepancy indicates that the impact of these factors might diminish over time [48] and highlights the need for further research to comprehensively understand these dynamics. To the best of our knowledge, the prognostic value of health literacy for high societal costs has not been previously documented.

The main limitation of the current study is that it’s powered on the primary outcome of the RCT, not on the outcomes under consideration. This limited our ability to adjust for covariates. Therefore, we prioritized adjustment for the same core set of covariates as in the identification study, along with group allocation, precluding additional adjustment for the other modifiable prognostic factors, which may have affected the observed associations. Furthermore, we did not assess other potentially important prognostic factors of high societal costs, such as health behaviors, physical demands, or claim-related factors. A second potential limitation is the absence of correction for multiple testing and the lack of assessment regarding whether our results would remain statistically significance after such adjustment. This approach was chosen to align our methodology with the identification study, which did not implement this correction. Moreover, to the best of our knowledge, there is no general consensus on when, and if so, how, adjustment for multiple hypotheses should be applied [59]. A third potential limitation is the likelihood of underestimating the true value of both healthcare utilization and productivity loss. We lack data on medication use, private healthcare utilization, and productivity loss related to reduced productivity while at paid work (presenteeism) and unpaid work. However, we deem the impact of this limitation to be of only minor importance in the current study, as healthcare utilization and productivity loss was measured and valued consistently with the approach used in the identification study, and equally for all participants. A fourth potential limitation is the lack of detailed information in the registers, which prevented us from differentiating between the cause leading to healthcare utilization. Consequently, some of the included costs related to healthcare utilization could potentially stem from reasons other than musculoskeletal disorders.

The main strength of the current study is that it was conducted as a replication study in accordance with the PROGRESS framework [12, 16], pre-planned with a published statistical analysis plan [60], and reported following the REMARK guidelines [19]. Furthermore, the study estimates the prognostic value of modifiable prognostic factors beyond a core set of covariates. Moreover, there was a low volume of missing data for the prognostic factor variables, and no missing data for variables used to calculate the outcome scores. Finally, previous research [22] confirm representativeness of the study sample in terms of age, sex and occupation compared to all eligible candidates.

## Conclusion

In conclusion, this study replicated low return-to-work expectancy as a statistically significant modifiable prognostic factor associated with high societal costs among people on sick leave (≥ 7 consecutive weeks) due to musculoskeletal disorders. Similar results were observed when high costs were separately related to healthcare utilization and productivity loss. These findings contribute to the ongoing research into clinical and welfare pathways, highlighting return-to-work expectancy as a potential target area for intervention which could reduce high societal costs among people on sick leave due to musculoskeletal disorders.

## Supplementary Information

Supplementary Material 1.

## Data Availability

Anonymized individual participant data (including data dictionary) will be available upon reasonable requests (addressed to mgrotle@oslomet.no), to researchers who provide a methodologically sound scientific proposal that has been approved by an ethics committee and by the scientific board of the MI-NAV study.

## Abbreviations

BCa: Bias-corrected and accelerated
CI: Confidence Interval
EPP: Events per parameter
EQ-5D-5L: EuroQol 5 dimensions
GP: General Practitioner
ICPC-2: International Classification of Primary Care 2ed edition
KPR: The Municipal Patient and User Registry
MI: Motivational interviewing
MSK-HQ: Musculoskeletal Health Questionnaire
NAV: The Norwegian Labour and Welfare Administration
NOK: Norwegian krone
NPR: The Norwegian Patient Registry
NRS: Numeric Rating Scale
OR: Odds ratio
PROGRESS: PROGnosis RESearch Strategy
Q: Question number
RCT: Randomised controlled trial
REMARK: REporting recommendations for tumor MARKer prognostic studies
STarT MSK: Keele STarT MSK Tool
SVAI: Stratified vocational advice intervention
TSD: The Service for sensitive data
UC: Usual case management
WP: Work package
ÖMPSQ-SF: The Örebro Musculoskeletal Pain Screening Questionnaire Short Form
€: Euros
