# Supplementary material for: Modifiable prognostic factors of high societal costs among people on sick leave due to musculoskeletal disorders: a replication study
Source: BMC Musculoskelet Disord. 2024 Dec 3;25:990. doi: 10.1186/s12891-024-08132-3 (PMC11613927; doi:10.1186/s12891-024-08132-3)
Supplement: Supplementary file 1 — Supplementary Material 1. [file 12891_2024_8132_MOESM1_ESM.pdf]

Additional file 1:

Table A1. Sensitivity analysis where the two outliers for high cost related to healthcare utilization are removed. Binary logistic regression analyses; individual associations between modifiable prognostic factors and high cost related to healthcare utilization (n=507)

	**High costs related to healthcare utilization**	
	Crude OR (95% CI)	Adjusted OR* (95% CI)
Pain severity (NRS, 0-10)	1.00 (0.91-1.11)	1.04 (0.93-1.16)
Self-perceived health (EQ-5D-5L, EQ VAS, 0-10)	1.07 (0.97-1.19)	1.08 (0.97-1.21)
Depressive symptoms (ÖMPSQ-SF, Q6, 0-10)	1.01 (0.94-1.08)	1.02 (0.95-1.10)
Sleep quality (ÖMPSQ-SF, Q4, 0-10)[a]	1.05 (0.97-1.13)	1.05 (0.97-1.14)
Return to work expectancy (ÖMPSQ-SF, Q8, 0-10)	1.08 (1.01-1.16)	1.09 (1.00-1.18)
Work satisfaction (0-10)	0.88 (0.80-0.97)	0.89 (0.81-0.99)
Disability (EQ-5D-5L, Q3) (ref: no/slight problems)		
Moderate problems	1.18 (0.73-1.90)	1.20 (0.73-1.99)
Severe problems/unable to do	1.25 (0.75-2.08)	1.14 (0.67-1.95)
Health literacy (MSK-HQ, Q12) (ref: completely/very well understanding)		
Moderate understanding	0.79 (0.49-1.28)	0.88 (0.53-1.45)
Slightly/no understanding	0.69 (0.36-1.32)	0.71 (0.35-1.45)
Long-lasting disorder expectation (STarT MSK, Q6) (ref: no)	1.45 (0.84-2.51)	1.43 (0.79-2.62)

EQ-5D-5L indicates EuroQol 5 dimensions; CI, confidence interval; MSK-HQ, Musculoskeletal Health Questionnaire; NRS, Numeric Rating Scale; OR, odd ratio; ÖMPSQ-SF, Örebro Musculoskeletal Pain Screening Questionnaire Short Form; STarT MSK, Keele STarT MSK tool; Q, question number. [a]Fractional polynomial function = Sleep quality -4.5914. *Adjusted by sex, age, education level, absenteeism related diagnosis type, pain duration, group allocation, and costs related to healthcare utilization prior to inclusion.

Table A2. Sensitivity analysis where the covariate "cost related healthcare utilization prior to inclusion" is modelled with fractional polynomials 1. Binary logistic regression analyses; individual associations between modifiable prognostic factors and high cost related to healthcare utilization (n=509)

	**High costs related to healthcare utilization**
	Adjusted OR* (95% CI)
Pain severity (NRS, 0-10)	1.06 (0.95-1.18)
Self-perceived health (EQ-5D-5L, EQ VAS, 0-10)	1.07 (0.95-1.19)
Depressive symptoms (ÖMPSQ-SF, Q6, 0-10)	1.02 (0.95-1.09)
Sleep quality (ÖMPSQ-SF, Q4, 0-10)[a]	1.06 (0.98-1.15)
Return to work expectancy (ÖMPSQ-SF, Q8, 0-10)	1.10 (1.02-1.20)
Work satisfaction (0-10)	0.89 (0.81-0.99)
Disability (EQ-5D-5L, Q3) (ref: no/slight problems)	
Moderate problems	1.20 (0.72-1.99)
Severe problems/unable to do	1.05 (0.61-1.80)
Health literacy (MSK-HQ, Q12) (ref: completely/very well understanding)	
Moderate understanding	0.91 (0.55-1.51)
Slightly/no understanding	0.74 (0.37-1.50)
Long-lasting disorder expectation (STarT MSK, Q6) (ref: no)	1.57 (0.85-2.89)

EQ-5D-5L indicates EuroQol 5 dimensions; CI, confidence interval; MSK-HQ, Musculoskeletal Health Questionnaire; NRS, Numeric Rating Scale; OR, odd ratio; ÖMPSQ-SF, Örebro Musculoskeletal Pain Screening Questionnaire Short Form; STarT MSK, Keele STarT MSK tool; Q, question number. [a]Fractional polynomial function = Sleep quality -4.5914. *Adjusted by sex, age, education level, absenteeism related diagnosis type, pain duration, group allocation, and costs related to healthcare utilization prior to inclusion (fractional polynomial function = costs related to healthcare utilization prior to inclusion^(0 0).
